# English Lunacy in 1890

**Published:** 1891-08-15

**Authors:** 


A Weekly Journal of

Science, Medicine, Nursing, and Philanthropy.

*For Hospitals, Asylums, Infirmaries, Dispensaries, Medical Practitioners, Students, Nurses, and the Charitable Public.*

Vol. X.—No. 255.] [Aug. 15, 1891.

### English Lunacy in 1890.

Year by year the Blue Book of the Lunacy Commissioners makes its appearance, but as it never sees the light before Midsummer, we are always six months behind in our knowledge of the exact number of our mad folk, and of the other statistical information supplied by this volume. This information is appalling enough. Eighty-six thousand odd of our countrymen were, on the first of January, 1891, known to be of unsound mind, and this total, alarming as it is, is probably far short of the real truth. It shows an increase, too, of 728 during the year, and although this increase is considerably below the average of former years, we fear we cannot extract from the fact any ray of hope. In every ten thousand of the population there are twenty-nine insane persons, and it must be noted that this proportion refers only to those known to be insane at the time of compilation of the statistics. During 1890 there were upwards of nineteen thousand lunatics admitted to our various public and private asylums, and upwards of eleven thousand were discharged therefrom, so that our readers can form some idea of the prevalence of this sad disease.

The report bears evidence of the great care now shown to the insane by all classes of asylum officials. This is conclusively proved by the fact that although an immense number of the insane are suicidally disposed only fourteen succeeded in committing the act while in asylums, and it is curious to notice that ten of the fourteen were men.

It would seem that recoveries are in asylums always calculated on the admissions, and the 6,250 cases recovering during the year 1890 would yield a percentage of 38·59, which is just a fraction lower than the recovery rate for 1889.

The Commissioners refer to those counties in which the accommodation for the insane poor is insufficient. They inform us that 3,317 lunatics belonging to the County of London are in asylums other than those of their own county, and that the "excess of cost to the ratepayers of London owing to this boarding out, would probably amount to about £45,000 per annum." A very good asylum can be built for £130 a bed, so that each year this system is allowed to continue, a sum is spent which would provide room for something like 350 patients, and be it remarked the asylum has to be built in the end, the ratepayers having to find the money twice over, so to speak. County Councils have in some instances set themselves against pensions, but here in one county is enough money spent in this boarding out plan to pension all the asylum officials in every county in England, and we are not aware that any ratepayer or councillor has raised his voice against it.

We have in these columns more than once pointed out the advisableness or rather absolute necessity of placing this pension question on a secure footing, and we are pleased to quote the following paragraph from the report under notice:—"Fair salaries or wages, with the prospect of liberal pensions after disablement or reasonable length of service, offer, we think, the most influential inducements to really suitable persons to enter asylum service, and to remain in it as a permanent occupation. . . . The work of the Medical Superintendent of an asylum is anxious, harrassing, and not unattended by personal risk. His responsibility is unceasing; and few can venture, without danger to health, to extend their tenure of the office beyond moderate limits. The work of the lower officials, who are much in contact with the insane, is also wearing and not free from danger; while it calls forth the exercise of qualities of intelligence, tact, and patience."

To those of us accustomed to hospital management only, one of the most remarkable statements in the volume is that relating to the cost of maintaining patients in our county and borough asylums. It is stated on page 64 that the average weekly cost per head is only 8s. 10⅞d., and if the small Borough Asylums be omitted the average is reduced to 8s. 7⅝d. And we learn from a table given on the same page that these sums include not only salaries of all the officers, medical and lay, but also clothing of the patients. Hospital Boards might well spend a little time in making themselves familiar with the management of asylums.

Many of the old asylums are being added to, and new ones are about to be built. There would seem to be no end to the want of accommodation for pauper lunatics; no cessation of the cry, "Still they come."

